# Excellence Across the Lifespan Champions: A Partnership With the National Center to Reframe Aging

**DOI:** 10.1093/geroni/igaf122.2146

**Published:** 2025-12-31

**Authors:** Sara Hart, Rebekah Perkins, Patricia D’Antonio, Katarina Friberg-Felsted, Kara Dassel, Jacqueline Eaton

**Affiliations:** University of Utah, Salt Lake City, Utah, United States; University of Utah, Salt Lake City, Utah, United States; Gerontological Society of America, Washington, District of Columbia, United States; University of Utah, Salt Lake City, Utah, United States; University of Utah, Salt Lake City, Utah, United States; University of Utah, Salt Lake City, Utah, United States

## Abstract

To promote age-friendly policies within a College of Nursing, the “Excellence Across the Lifespan Champions” program was developed in partnership with the National Center to Reframe Aging. This initiative aimed to cultivate a cohort of faculty and staff champions to lead institutional culture change. The program utilized a professional development framework to incentivize participation. Champions received education and mentorship while conducting age-inclusivity mini-projects aligned with their roles. Peer-mentored group publications were encouraged to foster collaboration and knowledge dissemination. Program design involved collaborative meetings with the Center and institutional partners. A synchronous online format was chosen, limited to six faculty and six staff to ensure project diversity. Recruitment targeted staff leadership teams and faculty through a custom flyer and interest survey. The program included four online training sessions followed by a 3-month project implementation phase, with individualized feedback and mentorship throughout. This innovative partnership demonstrates a commitment to professional development that challenges ageist attitudes and hidden biases while promoting age-inclusive practices. The “Excellence Across the Lifespan Champions” program serves as a model for organizations seeking to reframe aging and foster an age-friendly ecosystem. This session will detail champion projects and their early impact.

